# Some Tips for Writing Science

**DOI:** 10.1523/ENEURO.0497-22.2022

**Published:** 2022-12-21

**Authors:** Matteo Carandini

**Affiliations:** UCL Institute of Ophthalmology, University College London, London, WC1E 6BT, UK

When preparing a scientific paper, we typically write with coauthors who have different backgrounds and styles, and we target readers who have little time and patience. To help both readers and writers, some guidelines can be useful. These include centering the paper on the questions that it addresses, writing simply and briefly, structuring sentences so that new information comes at the end, using separate paragraphs for different points, summarizing each paragraph in its opening sentence, and making figures that minimize ink. These suggestions help make a paper easy to read.

## Introduction

Scientific ideas can be understood only if they are simple, and the job of making them simple is the writer’s, not the reader’s. Adding to the difficulties, scientific writing is often a collective effort, and many of us grew up writing different languages. For these reasons, it helps to identify some guidelines. Let’s start here in the first paragraph, which should set up the broad area of inquiry and ideally open with a vivid sentence. My favorite is by Barlow (1961): “A wing would be a most mystifying structure if one did not know that birds flew.” Wouldn’t you want to read the rest?

The rest of the Introduction is there to pose specific questions in the context of the literature and as objects of debate. These questions are the pillars of the paper: pose them in the Introduction, answer them in Results, and discuss your answers in Discussion.

Identifying these questions takes surprising effort. We think we already know what questions our work addresses, but most likely in the myriad decisions we made during the work we followed gut feelings. When writing, those feelings need to move from the gut to the language areas of the cortex. Moreover, each piece of evidence can answer multiple questions, so the choice of questions goes hand in hand with the choice of evidence to include. In the end, the paper may end up addressing different questions from the ones that we posed at the beginning of the study.

When presenting the questions, beware of asserting that they are “interesting,” “important,” or “understudied.” Let the reader conclude that they are interesting or important. As for “understudied,” it makes a weak case: they may be understudied because unimportant.

The last paragraph of the Introduction usually describes the main results. Mention the methods, especially if they are novel or provide new avenues into a problem, and summarize the take-home messages. In this essay, these are: write simply, identify key questions, write in paragraphs, use topic sentences, structure the sentences with information in the right places, and make figures with little ink. Strive for brevity, ideally keeping the Introduction below 500 words. (This one is below 400 words.)

## Methods

Regardless of where a journal places the Methods section, write so that readers can go directly from Introduction to Results. This means distributing brief descriptions of the methods across Results. And if you are writing a methods paper, consider merging the two sections: “Methods and Results.”

Conversely, you can safely assume that people know that methods are in Methods, so it rarely makes sense to write “see Methods.” An exception is if Methods contain results, such as pilot measurements or a mathematical derivation, and you want the readers to know they are there.

Unless you are writing for *Nature*, use American English: it is the language of science, and international scientists are used to it. They are typically not used to British English; they will think there are typos and be distracted.

Use the full power of your word processor and of your citation manager: it might sound like more work but it’s not, and it dramatically reduces errors. Use your word processor to indicate headings, number figures, insert captions, refer to figures, etc. If possible, tell it to highlight fields, so you see which text is automatically generated. And never enter citations by hand: use your citation manager.

## Results

The first paragraph of Results typically lays out a brief oadmap of how results are organized. It can also summarize methods that apply to the whole study. The rest of Results is often divided into subsections, each of which ideally has a figure with the same title.

### Style

The key to a scientific paper is to be as simple and brief as possible, because our readers are often rushed. Especially the reviewers. Moreover, being simple and brief helps write collaboratively, which is a necessity as most papers have multiple authors.

For brevity, we must “omit needless words” as advised in the classic booklet by Strunk and White (1959). After writing, we go through every word and ask if cutting it changes the meaning. If it does not, we cut it. This is painful, so it requires motivation. It is easiest after acceptance, when one no longer worries about reviewers and can concentrate on fitting with the journal guidelines.

Some words can be removed reflexively. These include “respectively” because it is generally obvious; “recent” because it ages quickly; “very” because it often reduces rather than amplify (also in French: “je l’aime beaucoup” is weaker than “je l’aime”).

The average scientific paper uses too many passive voices, generic verbs, and nouns. Instead of “the implantation of the widget was performed,” write “we implanted the widget.” It is briefer, it is active instead of passive, it explains who did what, and it replaces a generic verb (“to perform”) and a noun (“implantation”) with an informative and direct verb (“to implant”). Another example is “A is dependent on B”: it is better to write “A depends on B,” because it is active and uses a stronger verb: “to depend” versus “to be.”

This said, the passive voice is invaluable when the important subject is not the one acting (Strunk and White, 1959; Pinker, 2015). For example, “the vaccine was discovered in 2035” focuses on the vaccine, whereas “scientists discovered the vaccine in 2035” brings scientists into the picture, which may be irrelevant in the argument (Freeman, 2013). The passive form can be good, but it should be a conscious decision.

While much of this advice is general, there is also a specific requirement for scientific writing: avoid synonyms (Derrington, 2015b; Pinker, 2015). Synonyms confuse the reader, especially those who are not specialists (e.g., someone reviewing your grant). Once you have named something with one or more words, use those words consistently throughout. This may clash with our education: we were taught to vary our words, perhaps to show off. This is not that game. The game here is to ensure people follow our logic with minimal effort.

### Sentences

The best way to improve writing is to learn how to structure a sentence. When I started in science, I thought I already knew how to write a sentence, and yet I did not know that a sentence has different places for new information and old information. As explained in a lovely article titled *The science of scientific writing* (Gopen and Swan, 1990), this is often the single but devastating reason why scientific prose is impenetrable.

A sentence is much easier to understand if its beginning relates to previous information, and new information appears at the end, where readers expect it and are receptive to it. Unless this is already obvious to you, read Gopen and Swan (1990), and inspect their two examples of impenetrable paragraphs. Those paragraphs suddenly become clear when each sentence is rearranged to put old information at the beginning and new information at the end.

### Paragraphs

The unit of text in a scientific paper is the paragraph, and it greatly helps if each paragraph makes a distinct point and if this point is summarized in the first sentence. This sentence is called the “topic sentence” (Strunk and White, 1959). Writing in paragraphs might not be obvious to people coming from other languages (it was not to me). Moreover, summarizing the contents at the start of each paragraph will strike some as “giving away” the result. Ignore these concerns.

A topic sentence will greatly help the readers: it motivates them to read the rest of a paragraph and guides them in interpreting it. Or, if they are in a hurry, it allows them to skip the rest of the paragraph while knowing roughly what they skipped.

Amazingly, topic sentences will also help the writers. Indeed, if a paragraph covers multiple topics and makes multiple points, we will be unable to summarize it with a reasonable topic sentence. That is our cue that the paragraph needs to be split or otherwise reorganized.

When the topic sentences are done right, putting them in sequence should provide a summary of the paper. This could be our first draft of the Abstract. It could also be our first draft of the paper: we could start by writing all the topic sentences and then flesh out the rest. This method is particularly useful when writing collaboratively, as it signals to all coauthors the flow of the argument and what goes where. In fact, I typically put all the topic sentences in bold until the paper is ready.

The style of writing is sometimes called “assert/justify”: first we assert something and then, for the readers who care or who disagree, we support it with evidence. As explained by Derrington in his blog (Derrington, 2015a), this style is ideally suited both to readers who are distracted and rushed (think of reviewers), and to readers who care deeply and want to know the details (think of your closest colleagues). As a bonus, it makes it easier to write well. This and other insights by Derrington may also be found in his book (Aldridge and Derrington, 2012).

Some people, including some excellent scientists, structure their paragraphs in the opposite way: first all the facts and then a last sentence with the take-home message. I do not recommend this style because it assumes tremendous commitment from the reader. Perhaps it is okay in a landmark paper, where the readers are hanging on every word. But it would be a mistake when writing for rushed readers, and especially in a grant application. Some people, finally, think each paragraph should make its point twice: at the start and the end. I find that verbose: it is enough to do it once, at the start.

### Figures

Strive to organize your figures so that they tell the whole story. Think about someone using your figures for a “journal club” presentation. Will they have what they need to tell your story?

Within a figure, one generally proceeds from the exemplar to the general. For instance, in neurophysiology, the first panels might illustrate the activity of example neurons (ideally chosen based on objective criteria such as the quantiles of a distribution), the subsequent ones might show the activity of a population of neurons in a single session, and the final ones might summarize this activity across sessions.

Figures are a fundamental aid, but they are not the object of the research. So, it is not advisable to make them the subject of a sentence (“Fig. 1 shows that the brain is wet”). To help keep the focus on the object of the research, I refer to figures only in parenthesis: “The brain is wet (Fig. 1).”

Make sure the text walks the reader through each panel in each figure. If a figure or panel does not need to be mentioned, does it need to be in the paper at all? Perhaps it can go to Supplementary Materials. These, by the way, are supplementary; do not demand that your readers look at them.

Make the figures to final size and scale them at 100% in your paper. This way you will be sure that they fit in a page of the journal, and that the font sizes and other conventions are consistent. Also, when it is time to publish, it is simpler to ask the journal to scale all figures equally.

As for the contents of the figures, this is not the place for detailed suggestions, but the golden rule is to “minimize ink.” This rule comes from the classic *The visual display of quantitative information* (Tufte, 1983), which is fun and has some great suggestions. It means that ink is useful only if it conveys information. The remaining ink masks this information and should be removed. Examples of this include boxes, grids, intrusive or unnecessary axes, panels with dark backgrounds. Another source of masking is crowding: reduce it by putting space between panels and between data and axes. Conversely, do use ink for things that you want people to see. For instance, do not plot yellow curves on a white page: they are invisible.

When choosing a colormap, choose one that has the dimensionality of the data and emphasizes regions where signals are strong. For instance, if the data are one-dimensional, choose a one-dimensional colormap (e.g., light to dark), not a two-dimensional one (varying e.g., both in hue and darkness). Also, given that the background of a page is white, choose a colormap where low signal is light and high signal is dark. Avoid colormaps that have discontinuities (e.g., a yellow fine band between green and red), because they provide a salient contour at an arbitrary value. If you want contours, add contours.

Regarding data images: if you do anything to an image, you must do it to the whole image. For instance, if you increase image contrast (a fine idea), you must do it to the whole image, not just a portion. If you absolutely must do something only in a region of the image (e.g., set activity to zero outside the brain or in an obvious artifact) indicate where you did this with a special color.

### Numbers

It is hard to read prose that is constantly interrupted by numbers, confidence intervals, *p*-values, statistical tests, and the like. These can often be moved into tables, legends, figures, and Methods. It also helps to keep them tight by choosing a reasonable level of precision (like 61% instead of 61.37%) and keeping it consistent (like 0.1 and 6.0 rather than 0.1374 and 6).

### Sequence

Which sections shall we write first? Personally, I prefer to start by drafting title and Introduction, because it forces me to think hard about the questions at hand and the data that we must include to answer them. Others, instead, prefer to write Results first, or to make all the figures first and write the captions. As with much of what I have covered, it is a matter of personal taste.

## Discussion

The first paragraph of Discussion usually summarizes again the main findings. Here, I have suggested centering a paper on the questions that it addresses, being simple and brief, structuring sentences with the old before the new, separating points in paragraphs that start with a topic sentence, and making figures that minimize ink.

This said, many people do not read the Discussion, so make sure that your story can be understood from Introduction and Results. In fact, ideally it should be possible to understand your paper just from title, Abstract, and figures.

The rest of Discussion should return to the questions raised in Introduction and discuss how the Results addressed and hopefully resolved those questions. It is the place to be most scholarly in relating the present work with the literature. Once again, beware of synonyms: use the same words as in Introduction and Results.

The key job of the Discussion is to draw conclusions, which are different from summaries. Summaries concern the specifics of experiments, procedures, and results. Conclusions are at a higher level, which is independent of those specifics. For instance, in a neuroscience paper the procedures may involve brain recordings and manipulations, and the conclusions would be about how the brain works.

Finally, the Discussion is an opportunity to indicate the limitations of the paper. A key limitation here is that I have barely mentioned grant proposals. This would require an essay of its own, or even a book (Aldridge and Derrington, 2012). The main advice is to put most effort into the first page, typically called Specific Aims. Arguably, this page decides whether the grant is won or lost, and the rest of the proposal can only disappoint. A great trick is to summarize it in “10 key sentences” (Derrington, 2014) indicating: (1) what the project will achieve; (2) why this matters; (3–5) the three aims of the project, at the level of questions and hypotheses; (6) the general approach; (7–9) the three aims, at the level of specific approaches; (10) how the world will be different after the work is done. These sentences will then be the core of the proposal and be reused throughout.

As scientists we may not feel that we are writers, but ultimately our only products are scientific writings and the accompanying figures. It is thus essential that we write well, and that we teach our trainees to write well. Fortunately, while there are many simple ways to get papers wrong, there are also many simple ways to get them right, and hopefully this document will have helped identify some of these ways.

### Acknowledging people and grants

This section is for thanking the people who helped with the work and the grants that funded it. When thanking people, remember to first ask them for permission. It is also best to specify author contributions, where possible using the CredIt taxonomy (https://credit.niso.org/), and ideally to express the results in a table if the journal allows it.

## Synthesis

Reviewing Editor: Christophe Bernard, INSERM & Institut de Neurosciences des Systèmes Decisions are customarily a result of the Reviewing Editor and the peer reviewers coming together and discussing their recommendations until a consensus is reached. When revisions are invited, a fact-based synthesis statement explaining their decision and outlining what is needed to prepare a revision will be listed below. The following reviewer(s) agreed to reveal their identity: NONE.

na
